# Determinants of change in fertility pattern among women in Uganda during the period 2006–2011

**DOI:** 10.1186/s40738-018-0049-1

**Published:** 2018-06-27

**Authors:** Paulino Ariho, Allen Kabagenyi, Abel Nzabona

**Affiliations:** 1 Department of Population Studies, School of Statistics & Planning, College of Business and Management Sciences, Makerere University, P.O.Box 7062, Kampala, Uganda; 2 Department of Sociology and Social Administration, Kyambogo University, P.O.Box 1, Kyambogo, Uganda

**Keywords:** Change in fertility, Children ever-born, Decomposition, Socioeconomic factors, Demographic factors, Uganda

## Abstract

**Background:**

Studies on fertility in Uganda have attributed fertility reduction to a shift in the overall characteristics of women of reproductive age. It is not clear whether the reduction in fertility is due to changing socioeconomic and demographic characteristics over time or stems from the shifts in the reproductive behavior of women. In this paper we examine how fertility rates have changed between 2006 and 2011 and whether these changes have resulted from changing characteristics or from changing reproductive behavior of women.

**Methods:**

Using the 2006 and 2011 Demographic and Health Survey data for Uganda, Multivariate Poisson Decomposition techniques were applied to evaluate observed changes in fertility.

**Results:**

Changing characteristics of women aged 15–49 years significantly contributed to the overall change in fertility from 2006 to 2011. The change observed in older age at first marriage was the major contributor to the changes in fertility. The contribution that can be attributed to changes in reproductive behavior was not significant.

**Conclusions:**

This study finds that the major contribution to the reduction in fertility between 2006 and 2011 was from increased education and delayed marriage among women. Continued improvement in secondary school completion, will lead to older age at first marriage and will continue to be an important factor in Uganda’s declining fertility rates.

## Background

Total fertility rate (TFR), the number of live births that a woman would have at the end of her reproductive years if the prevailing age specific fertility rates remained constant [1] has declined in all developing regions of the world. In Asia and Latin America and sub-Saharan Africa, a decline in TFR began in the mid-1970s, and 1990s respectively [2]. Whereas Asia and Latin America have had rapid fertility declines, the declines in Africa and particularly Sub-Saharan Africa have been modest [3]. For example between 1950 and 2010, total fertility rates (TFR) in Asia and Latin America declined from 5.8 and 5.9 children per woman respectively to about 2.3 children per woman, while Africa’s fertility declined from 6.6 to 4.9 children per woman in the same period [4]. Kabagenyi, et al. [5] examined the onset of fertility transition in Uganda but found no evidence of a stall. They indicated that Uganda’s TFR declined for a period of time and then remained constant. The TFR ranged between 8 in 1970s to 6 in 2010. Current population census results in Uganda indicate that between 2002 and 2014, TFR declined from 7.0 children per woman to 5.8 children per woman [6]. However, the TFR of 5.8 children per woman was the tenth highest in the world [7].

Studies have found fertility changes to be associated with changes in demographic, socioeconomic and cultural factors which particularly influence family size, contraceptive access and use and age at first marriage [8–10]. Changes in fertility arise from changing characteristics of women as well as changing reproductive behavior that occur as a result of changing characteristics [9]. The changing characteristics of women and changing reproductive behavior can have varying influences on fertility. For instance, Rutayisire, et al., [9] found that a decrease in the proportion of women who were currently in unions (marriage and cohabitation) contributed much to lowering Rwanda’s fertility between 1992 and 2000. On the other hand, changing reproductive behavior revealed that the fertility of the women in unions was higher in 2000 compared to 1992. The decrease in the proportion of women in unions was more than offset by the shift in their reproductive behaviors and thus fertility remained higher [9].

Uganda’s persistently high fertility has been attributed to low levels of contraception among women [11]. Previous studies in Uganda have documented differential changes in fertility among population sub groups. For instance faster decline is shown among the most educated women; these reside in urban areas and specific regions of the country [12, 13]. These studies have highlighted the influence of women’s social, economic and demographic factors in fertility change. The studies however have not isolated the change in fertility that is attributable to changing characteristics of women over time from that which is due to the changing reproductive behavior.

Changing characteristics refer to changes in proportion of the population with particular social, economic and demographic characteristics, while the change in reproductive behavior refers to the changes in the behavior of the population as a result of the change in characteristics [14]. In this study we used number of children ever born (CEB) as the measure fertility to conduct a decomposition analysis of change in fertility in Uganda between 2006 and 2011. The CEB measures the number of children born to a woman reported up to the moment at which the data are collected [1]. Specifically, this study determines variations in fertility between 2006 and 2011 that can be attributed to changing characteristics of women aged 15–49 years and assesses the variations that can be attributed to changes in reproductive behaviors of the women aged 15–49 years.

## Methods

Data obtained from the Uganda Demographic and Health Surveys (UDHS) conducted in 2006 and 2011 were used. Surveys were conducted by the Uganda Bureau of Statistics. The surveys were nationally representative cross sectional surveys that collected comparable demographic and health data on women aged 15–49 in the survey periods. The samples were obtained using a two-stage cluster sampling process beginning with the selection of clusters, or enumeration areas, followed by the selection of households from each cluster [15]. Since this study used secondary data, research approval from the Institutional Review Board was not applicable. The DHS data is freely available and the public can access it upon a formal request. We submitted an abstract to Measure DHS seeking permission to use the data and the required access was subsequently permitted.

This study is based on the secondary analysis of data of women aged 15–49 years collected in the 2006 and 2011 demographic and health surveys. The women aged 15–49 years in both the 2006 and 2011 surveys were asked about their birth histories and this provided information on the total number of children ever born. Since we were interested in determining the factors that may explain variations in fertility between 2006 and 2011, we adopted a multivariate decomposition analysis. Multivariate decomposition analysis is used to quantify changes over time into components attributable to changing characteristics and changes in reproductive behavior [14]. We used CEB as the measure of fertility.

### Inclusion and exclusion criteria

Women who had ever had sex were included since they were the only ones with the potential for pregnancy and child birth. In the DHS, women were asked *“how old were you when you had sexual intercourse for the very first time?”* This question was about the sexual activity of women. It is however possible that there was underreporting and misreporting, as the question may be sensitive to young women and especially unmarried adolescents who might not feel comfortable to disclose information related to sexual activities. It is thus possible that some women who had sex but did not declare so were excluded.

**Fig. 1:**
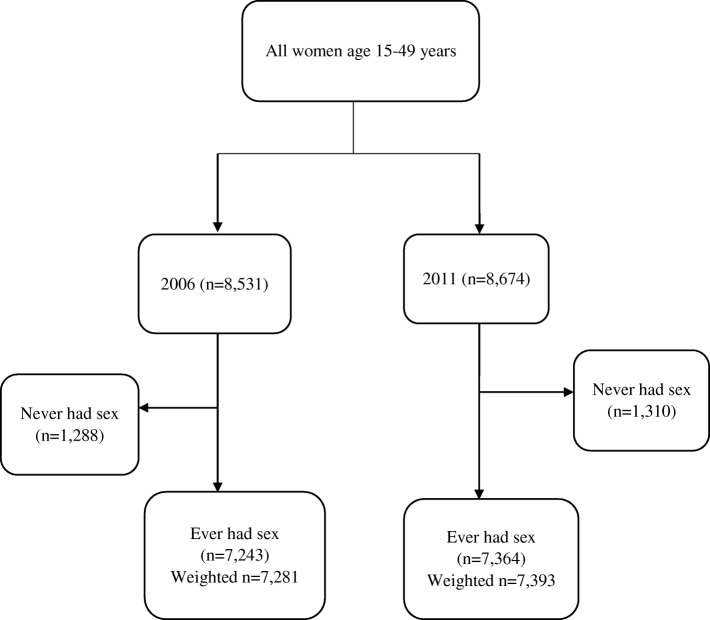
Derivation of the study sample

From a total of 8531 and 8674 women aged 15–49 years interviewed in the 2006 and 2011 UDHS respectively, we selected a total of 7243 and 7364 women aged 15–49 years who reported having ever had sex in the respective surveys. The weighted sample was 7281 women and 7393 women in 2006 and 2011 respectively. Figure 1 below is a flow chart that shows how the sample was derived.

### Variables

The dependent variable used in the study was the number of children ever born (CEB) to a female respondent in the two surveys. The independent variables were; age (current age of respondent in 5-year groups), education (highest education level attained by the respondent), residence (type of place of residence of respondent), religion (religion of respondent), wealth quintile (household wealth index), sex of household head (sex of the head of the household), polygamy (whether the respondent is in a polygamous union or was aware of other co-wives), working status of women (whether the respondent is currently working or not), exposure to family planning messages (whether respondent heard about FP on radio, TV and newspaper or not), knowledge about contraceptives (whether respondent has knowledge of any family planning method or not), source of contraceptive (the last source of modern family planning methods for users or respondent is non-user), age at first sex (age of the respondent at first sexual intercourse), ideal family size (Ideal number of children the respondent would have liked to have irrespective of number she already has), contraceptive use (current use of any contraceptive method), age at first marriage (age at first start of marriage or union. Due to the tendency to give birth before marriage, we introduced the “not yet married” category). These independent variables have effects on the outcome in two ways: the distribution effect (changes in characteristics of women) and the behavior effect (this is shown by regression coefficients of the variables on the outcome. For instance, change in fertility may be due to differences in distribution of women by education level attainment and also due to the effects of education on the reproductive behavior. Assuming women with higher education have a lower CEB, a decrease in TFR may be observed if more women in 2011 had a higher level of education when compared to women in 2006. This is an example of how changing characteristics can influence the TFR. In addition, women achieving a higher level of education may purposefully decrease the number of CEB, which would also lead to a decrease in TFR. Therefore changing reproductive behavior can influence the TFR.

### Data analysis

Using the 2006 and 2011 DHS data sets for Uganda, a Poisson regression offset by the natural logarithm of the current age of women was done for each survey period to determine the factors associated with number of children ever born. The data were first weighted to ensure representativeness of the sampled data. A weighting variable generated using the sample weight variable in the DHS data was applied in all statistical commands. This variable was used in all the models. The weighting took into account the complex sample design used in the DHS**.** The coefficients were exponentiated to yield incident rate ratio (IRR) to ease interpretation of the results. The incident rate ratio explains how changes in X (independent variable) affects the rate at which Y (CEB) occurs.

1 $$ \mathrm{In}\left({\mu}_i\right)=a+{X}_i{\beta}_i+\mathrm{In}(age) $$

where, *μ*_*i*_is the expected number of children born to a respondent based on the respondent’s demographic and socioeconomic characteristics; *X*_*i*_ are independent variables; *α* is a constant and *β*_*i*_ represents coefficients associated with the independent variables. ln(*age*), is the offset variable. This offset variable was generated from the variable age. In addition, we adjusted for marital status since marital status significantly related to the number of children ever born, as married or formerly married women are more likely to have more children ever born than the never marrieds. To know whether there was a significant change in number of children ever born between the two survey years, a one-way analysis of variance (ANOVA) which reports mean number of children ever born (MCEB) was used.

To determine the factors associated with the change in fertility between 2006 and 2011, a multivariate Poisson decomposition model was used to partition change in mean number of children ever born into components attributable to changing characteristics of women and that due to changing reproductive behavior of the women. The technique also partitions the two components into portions that represent the unique contribution of each predictor to each of the two components in a detailed decomposition (Powers, Yoshioka, & Yun, 2011). The general decomposition equation is as below:

2 $$ \overline{Y_B}-\overline{Y_A}=\overline{F\left({X}_A{\beta}_A\right)}-\overline{F\left({X}_B{\beta}_B\right)} $$

The eq. 2 can be further decomposed to eq. 3 below

3 $$ \overline{Y_B}-\overline{Y_A}=\left\{\overline{F\left({X}_A{\beta}_A\right)}-\overline{F\left({X}_B{\beta}_A\right)}\right\}+\left\{\overline{F\left({X}_B{\beta}_A\right)}-\overline{F\left({X}_B{\beta}_B\right)}\right\} $$

The summarized form of eq. 3 is as in eq. 4

4 $$ \overline{Y_B}-\overline{Y_A}=E+C $$

where; $\overline{Y_B}-\overline{Y_A}$ is the Mean difference in children ever born between Year B (2011) and year A (2006), *F*(*·*) is a logarithmic function mapping a linear combination of *X* (*Xβ*) to *Y,* X represents predictors and β represents regression coefficients. The summarized component E refers to the part of the change attributable to changing characteristics while the *C* component refers to the part of the change attributable to changing reproductive behavior. The Year 2011 is the comparison group and year 2006 is the reference group. *E* reflects the expected difference if Year 2011 were given Year 2006’s distribution of covariates. *C* reflects the expected difference if Year 2011 experienced Year 2006’s behavioral responses to *X*.

The results of the multivariate decomposition were interpreted using the coefficients on the two components. Specifically, a positive characteristics coefficient indicates the expected reduction in the fertility gap if the women in 2011 had the same distribution of characteristics of women in 2006. The fertility gap is means the change in number of children ever born. On the other hand, a negative behavioral effect coefficient indicates the expected increase in the fertility gap if women in the 2011 survey were given the coefficients of the 2006 survey. The overall percentage contribution of a characteristic to the gap in fertility is obtained by summing the percentages for the various categories of the characteristic. All the statistical significances of associations were determined at the 0.05 level of significance.

## Results

We studied a total weighted sample of 14,674 women aged 15–49 years, 49.6% of whom were from the 2006 survey and 50.4% from the 2011 survey. Our results indicate significant differences in characteristics that include age, education level, religion, current working status, polygamy and exposure to family planning messages (*p* < 0.05). Other characteristics that impacted CEB were source of modern family planning methods, knowledge of any family planning methods, contraceptive use, age at first sex, family size preference and age at first marriage. The characteristics of women and the associated difference in proportions in 2006 and 2011 are presented in Table 1.

**Table 1:** Distribution of women by selected characteristics in 2006 and 2011

	*n* = 7281	*n* = 7393		
Variable	2006 (%)	2011(%)	Difference (%)	*p*-value
Age				
15–19	833 (11.4)	923 (12.5)	1.1	0.004
20–24	1581 (21.7)	1495 (20.2)	−1.5	
25–29	1401 (19.2)	1558 (21.1)	1.8	
30–34	1215 (16.7)	1077 (14.6)	−2.1	
35–39	939 (12.9)	1025 (13.9)	1.0	
40–44	734 (10.1)	729 (9.9)	−0.2	
45–49	579 (8.0)	587 (7.9)	0.1	
Education level				
No education	1613 (22.2)	1088 (14.7)	−7.4	0.000
Primary	4269 (58.6)	4388 (59.3)	0.6	
Secondary+	1399 (19.2)	1924 (26.0)	6.8	
Place of residence				
Urban	1199 (16.5)	1458 (19.7)	3.2	0.243
Rural	6082 (83.5)	5935 (80.3)	−3.2	
Religion				
Catholic	3139 (43.1)	3026 (40.9)	−2.2	0.000
Protestant	2498 (34.3)	2189 (29.6)	−4.7	
Muslim	811 (11.1)	990 (13.4)	2.3	
Other	833 (11.4)	1188 (16.1)	4.6	
Wealth quintile				
Poorest	1385 (19.0)	1360 (18.4)	−0.6	0.774
Poorer	1442 (19.8)	1381 (18.7)	−1.1	
Middle	1375 (18.9)	1375 (18.6)	−0.3	
Richer	1368 (18.8)	1406 (19.0)	0.2	
Richest	1711 (23.5)	1872 (25.3)	1.8	
Sex of household head				
Male	5105 (70.1)	5175 (70.0)	−0.1	0.927
Female	2176 (29.9)	2218 (30.0)	0.1	
Current working status				
Not working	1061 (14.6)	1930 (26.1)	11.5	0.000
Working	6220 (85.4)	5463 (73.9)	−11.5	
Polygamy				
No co-wife	3699 (50.8)	3890 (52.6)	1.8	0.029
Has co-wife	1503 (20.6)	1335 (18.1)	−2.6	
Not sure	2079 (28.6)	2168 (29.3)	0.8	
Exposure to family planning messages				
No	7111 (97.7)	6879 (93.0)	−4.6	0.000
Yes	170 (2.3)	514 (7.0)	4.6	
Source of modern family planning methods				
Non user	5965 (81.9)	5610 (75.9)	−6.0	0.000
Government	458 (6.3)	831 (11.2)	5.0	
Private	858 (11.8)	952 (12.9)	1.1	
Knowledge of any family planning methods				
No knowledge	173 (2.4)	82 (1.1)	−1.3	0.001
Has knowledge	7108 (97.6)	7311 (98.9)	1.3	
Contraceptive use				
Not using	5610 (77.1)	5344 (72.3)	−4.8	0.000
Using	1671 (22.9)	2049 (27.7)	4.8	
Age at first sex				
Below 15	1465 (20.1)	1188 (16.1)	−4.1	0.000
15–19	4007 (55.0)	3483 (47.1)	−7.9	
20+	412 (5.7)	471 (6.4)	0.7	
Don’t know	1397 (19.2)	2252 (30.5)	11.3	
Family size preference				
0–2 Children	547 (7.5)	575 (7.8)	0.3	0.010
3–4 Children	2970 (40.8)	3290 (44.5)	3.7	
5+ Children	3764 (51.7)	3528 (47.7)	−4.0	
Marital status				
Single	778 (10.7)	837 (11.3)	0.6	0.548
Formerly married	1166 (16.0)	1139 (15.4)	−0.6	
Married	5337 (73.3)	5417 (73.3)	0.0	
Age at first marriage				
Not yet married	778 (10.7)	842 (11.4)	0.7	0.002
Below 15	1103 (15.2)	1086 (14.7)	−0.5	
15–19	4105 (56.4)	3095 (52.8)	−3.6	
20+	1295 (17.8)	1561 (21.1)	3.3	

Between 2006 and 2011, there was a significant difference (*p* = 0.004) in the percentage of women by age. Specifically, the percentage changes in the women aged 15–19, 20–24, 25–29, 30–34, 35–39, 40–44 and 45–49 were; 1.1, − 1.5, 1.8, − 2.1, 1.0, − 0.2 and 0.1 respectively. The percentage of women who had not attained any level of education decreased by 7.4%, while the proportion of women who had attained at least a secondary level of education increased by 6.8%. Similarly, the percentage of women living in urban areas increased by 3.3%. Regarding wealth quintile, there was slight reduction in the proportion of women in the poorest, poorer, and middle categories and a slight increase in proportion of women in the richer and richest categories. The results also indicate that the proportion of women in the “not working” category increased by 11.5%. There was a reduction of 2.5% in the proportion of women who were in a polygamous union.

The proportion of women reached by family planning messages increased by 4.6% in the period 2006–2011. Similarly, the proportion of women who reported getting modern family planning methods from the government facilities increased by 5%. There was a notable change in contraceptive use. The proportion of women who were currently using any contraceptive increased by 4.8% between the two survey periods. The findings further indicate that the proportion of women whose sexual debut was below the age of 20 years reduced by 12% signifying an increase in the age at sexual debut among women aged 15–49 years in 2011. Relatedly, there was a 3.7% reduction in the percentage of women marrying before the age of 20. The proportion of women who desired at least five children reduced by 4% and those who desired 3–4 children increased by 3.7%.

### Changes in fertility

Changes in fertility in this study are described by changes in number of children ever born (CEB) to the women. A computation of the mean number of children ever born (MCEB) was done using oneway ANOVA. The results revealed that the MCEB was 4.1 in 2006 and 3.9 in 2011 and that there was a statistically significant difference in MCEB between 2006 and 2011 (*p* = 0.000) at the 95% confidence level.

A Poisson regression offset by the natural logarithm of the current age of women was done for each survey period to find out the factors associated with CEB. Table 2 reveals that in both 2006 and 2011, the women who had attained at least a secondary level of education had a lower MCEB compared with their counterparts. Relatedly, in both 2006 and 2011, there was generally higher fertility among rural women compared to their urban counterparts. The results also indicate that women in the richest wealth quintile had a lower fertility. Female headed households had lower MCEB compared with the male headed households and for the two surveys, currently working women had higher fertility. Women who were in polygamous marriage in both 2006 and 2011 had higher MCEB compared with their counterparts who were not. When adjusted for age and marital status, the working status and co-wife status significantly affected number of children ever born only in 2006.

**Table 2:** Socio-economic and demographic factors associated with children ever-born in 2006 and 2011

Variable	2006 (*n* = 7243)				2011 (*n* = 7364)			
	IRR	95% CI	A(IRR)	A95%CI	IRR	95% CI	A(IRR)	A95%CI
Education level								
No education	1.00		1.00		1.00		1.00	
Primary	0.89***	0.86–0.91	1.03	0.99–1.06	0.84***	0.82–0.87	0.96***	0.93–0.99
Secondary +	0.52***	0.49–0.55	0.70***	0.67–0.73	0.51***	0.48–0.53	0.67***	0.64–0.70
Place of residence								
Urban	1.00		1.00		1.00		1.00	
Rural	1.49***	1.42–1.57	1.28***	1.23–1.34	1.57***	1.51–1.64	1.36***	1.31–1.41
Religion								
Catholic	1.00		1.00		1.00		1.00	
Protestant	0.99	0.96–1.02	1.01	0.98–1.04	0.99	0.95–1.02	0.98	0.95–1.02
Muslim	0.99	0.95–1.04	1.02	0.98–1.07	0.98	0.93–1.03	0.99	0.95–1.04
Other	0.98	0.94–1.03	0.99	0.94–1.03	0.99	0.94–1.04	1.00	0.96–1.04
Wealth quintile								
Poorest	1.00		1.00		1.00		1.00	
Poorer	0.98	0.94–1.02	0.98	0.95–1.02	0.96	0.92–1.01	0.96***	0.92–1.00
Middle	0.98	0.94–1.02	0.98	0.94–1.02	0.95***	0.91–1.00	0.94***	0.90–0.98
Richer	0.97	0.93–1.01	0.99	0.95–1.02	0.93***	0.89–0.97	0.92***	0.88–0.96
Richest	0.68***	0.65–0.71	0.77***	0.74–0.80	0.63***	0.60–0.66	0.69***	0.66–0.72
Sex of household head								
Male	1.00		1.00		1.00		1.00	
Female	0.90***	0.87–0.93	1.04***	1.00–1.07	0.87	0.84–0.90	1.03	0.99–1.07
Working status								
Not working	1.00		1.00		1.00		1.00	
Working	1.28***	1.22–1.35	1.14***	1.09–1.19	1.17***	1.12–1.21	1.05***	1.01–1.08
Polygamy								
No co-wife	1.00		1.00		1.00		1.00	
Has co-wife	1.05***	1.02–1.08	0.99	0.94–0.99	1.08***	1.05–1.12	1.03	1.00–1.07
Not sure	0.69***	0.66–072	1.39***	1.30–1.49	0.70***	0.67–0.73	1.39***	1.30–1.49
Exposure to family planning messages								
No	1.00		1.00		1.00		1.00	
Yes	0.57***	0.50–0.65	0.64***	0.57–0.72	0.60***	0.55–0.66	0.66***	0.61–0.71
Source of modern family planning methods								
Non user	1.00		1.00		1.00		1.00	
Government	1.17***	1.12–1.22	1.06***	1.02–1.10	1.17	1.13–1.22	1.08***	1.04–1.12
Private	0.89***	0.85–0.93	0.98	0.94–1.02	0.95	0.91–1.00	0.99	0.95–1.03
Knowledge of any family planning methods								
No knowledge	1.00		1.00		1.00		1.00	
Has knowledge	0.98	0.93–1.04	1.09***	1.03–1.16	0.97	0.87–1.08	1.04	0.93–1.15
Contraceptive use								
Not using	1.00		1.00		1.00		1.00	
Using	1.03	0.99–1.06	1.01	0.99–1.04	1.06***	1.03–1.10	1.02	0.99–1.05
Age at first sex								
< =14	1.00		1.00		1.00		1.00	
15–19	0.83***	0.81–0.86	0.85***	0.82–0.88	0.84***	0.81–0.88	0.88***	0.84–0.91
20+	0.62***	0.57–0.67	0.62***	0.58–0.66	0.54***	0.49–0.59	0.57***	0.52–0.62
Don’t know	0.99	0.95–1.03	0.90***	0.87–0.94	1.04	1.00–1.08	0.95***	0.91–0.99
Family size preference								
0–2 Children	1.00		1.00		1.00		1.00	
3–4 Children	1.39***	1.27–1.53	1.26***	1.16–1.36	1.20***	1.09–1.31	1.12***	1.03–1.22
5+ Children	2.01***	1.83–2.20	1.53***	1.42–1.65	1.77***	1.62–1.93	1.41***	1.307–1.53
Age at first marriage								
Not yet married	1.00		1.00		1.00		1.00	
< =14	6.40***	5.56–7.37	3.43***	2.94–4.01	7.67***	6.53–9.01	3.89***	3.26–4.64
15–19	5.48***	4.76–6.29	2.99***	2.56–3.49	6.22***	5.30–7.30	3.30***	2.78–3.93
20+	4.33***	3.75–4.99	2.26***	1.94–2.64	4.87***	4.14–5.73	2.50***	2.10–2.99

Note: *IRR* is Incident Rate Ratio. ***p < 0.05, AIRR: Adjusted IRR, A(95% CI):Adjusted 95% Confidence Interval. Results adjusted for marital status and current age of the women

After adjusting for age and marital status, women who knew any family planning methods in 2006 were found to have higher fertility compared with their counterparts who did not have any knowledge. Furthermore, women who were currently using a contraceptive method had higher fertility compared to those who were not. However, when adjusted for age and marital status, the influence of contraceptive use was not significant for the two surveys. In both 2006 and 2011, the MCEB was lower among women whose age at first sex was reported to be at least 20 years. Relatedly, the results indicate that in both 2006 and 2011, fertility was lower among women whose age at first marriage was 20 years or older. When adjusted for age and marital status; education, place of residence, wealth quintile, sex of household head, age at first sex and family size preference remained significant while contraceptive use was not significant. Table 2 shows both the crude and adjusted results.

### Decomposition of fertility change

Table 3 shows the overall contribution of characteristics and reproductive behavior of the women on the observed variation in number of children ever born. The findings indicate that the overall change in fertility between 2006 and 2011 was attributed to changing characteristics of women. Changing reproductive behavior did not contribute significantly to the observed change in fertility.

**Table 3:** Overall decomposition of change in number of children ever born

Components	Coefficient	95% CI	Percent (%)
E	−4.13***	−4.98-0.39	109.3
C	0.35	−1.96-2.66	−9.3
R	−3.78	−5.90-1.67	100

E = Component representing changing characteristics; C=Component representing changing reproductive behavior; ***p < 0.05

Results of the detailed decomposition presented in Table 4 reveal that the change in fertility was due to changes in age, education level, place of residence, wealth quintile, polygyny, household headship, exposure to family planning messages, contraceptive use, age at first sex, family size preference and age at first marriage.

**Table 4:** Detailed poisson decomposition of children ever born

Variable	Due to change in characteristics (E)				Due to change in coefficients (C)			
	Coef	Std. Err	*P*-value	%	Coef	Std. Err	*P*-value	%
Age								
15–19	1.000				1.000			
20–24	−0.961	0.075	*0.000*	25.4	0.038	1.339	0.977	−1.0
25–29	1.719	0.107	*0.000*	−45.4	0.249	1.999	0.901	−6.6
30–34	−2.309	0.138	*0.000*	61.1	−0.063	0.996	0.950	1.7
35–39	1.090	0.063	*0.000*	−28.8	0.190	1.444	0.895	−5.0
40–44	−0.249	0.014	*0.000*	6.6	−0.021	0.616	0.973	0.5
45–49	− 0.016	0.001	*0.000*	0.4	0.242	1.548	0.876	−6.4
Education level								
No education	1.000				1.000			
Primary	0.003	0.008	0.683	−0.1	1.030	6.220	0.868	−27.2
Secondary+	−0.646	0.143	*0.000*	17.1	−0.211	1.301	0.871	5.6
Place of residence								
Urban	1.000				1.000			
Rural	−0.283	0.053	*0.000*	7.5	1.376	8.141	0.866	−36.4
Wealth index								
Poorest	1.000				1.000			
Poorer	0.033	0.016	*0.037*	−0.9	0.066	0.549	0.904	−1.8
Middle	0.007	0.004	0.085	−0.2	− 0.185	1.191	0.877	4.9
Richer	−0.009	0.003	*0.007*	0.2	0.309	1.828	0.866	−8.2
Richest	−0.236	0.038	*0.000*	6.2	0.892	5.174	0.863	−23.6
Sex of household head								
Male	1.000				1.000			
Female	−0.006	0.001	*0.000*	0.1	1.082	6.368	0.865	−28.6
Current working status								
Not working	1.000				1.000			
Working	−0.109	0.140	0.436	2.9	−0.375	2.924	0.898	9.908
Polygamy								
No co-wife	1.000				1.000			
Has co-wife	−0.005	0.031	0.868	0.1	−0.597	3.531	0.866	15.8
Not sure	−0.069	0.013	*0.000*	1.8	−1.108	6.560	0.866	29.3
Exposure to family planning messages								
No	1.000				1.000			
Yes	−0.394	0.127	*0.002*	10.4	−0.112	0.655	0.865	3.0
Source of modern family planning methods								
Non user	1.000				1.000			
Government	−0.028	0.116	0.807	0.7	0.050	0.372	0.892	−1.3
Private	−0.024	0.026	0.353	0.6	−0.126	0.822	0.878	3.3
Contraceptive use								
Not using	1.000				1.000			
Using	0.301	0.105	*0.004*	−8.0	0.560	3.388	0.869	−14.8
Age at first sex								
Below 15	1.000				1.000			
15–19	0.391	0.119	*0.001*	−10.3	−0.394	2.518	0.876	10.4
20+	−0.167	0.024	*0.000*	4.4	0.374	2.212	0.866	−9.9
Don’t know	−0.587	0.178	*0.001*	15.5	−0.305	1.778	0.864	8.1
Family size preference								
0–2 Children	1.000				1.000			
3–4 Children	0.251	0.096	*0.009*	−6.6	0.915	5.640	0.871	−24.2
5+ Children	−0.688	0.110	*0.000*	18.2	0.712	4.771	0.881	−18.8
Age at first marriage								
Never married	1.000				1.000			
Below 15	−0.417	0.033	*0.000*	11.0	−1.468	8.345	0.860	38.8
15–19	−2.830	0.246	*0.000*	74.8	−3.842	21.673	0.859	101.6
20+	2.105	0.217	*0.000*	−55.7	−1.604	9.118	0.860	42.4
Constant					2.671	14.963	0.858	−70.6
Total	−4.133	0.432	*0.000*	109.3	0.351	1.179	0.766	−9.3

Note: Coef is the coefficient expressed in 1000.

The entries in italics are significant at *p*<0.05

The significant variables in the model (age, education level, place of residence, wealth index, sex of household head, polygyny, exposure to family planning messages, contraceptive use, age at first sex, family size preferences and age at first marriage) were tested for confounding. Age was found to be a confounder in the model. The findings indicated that when age was dropped from the model, education was the biggest contributor with 47.8%, followed by age at first marriage (40.9%), women’s preferred number of children (29.7%), working status (23.8%), contraceptive use (19.8%), exposure to family planning messages (12.7%), place of residence (11.1%), age at first sex (8.7%) and polygyny (3.4%).

## Discussion

Our analysis reveals that of characteristics and reproductive behavior, only changing characteristics of women significantly contributed to the observed change in number of CEB born between 2006 and 2011. The study results highlight importance of change in women’s educational attainment and age at first marriage to fertility. With an increase in the proportion of women who have attained at least a secondary level of education, this study highlights significant declines in fertility. The nationwide implementation of the Universal Secondary Education Policy in 2007 increased the proportion of women who attained at least secondary level of education in 2011. The increased attainment of higher level of education could have delayed entry into marriage and also increased the likelihood of using contraceptive methods. This confirms the notion that improvements in education of women is instrumental in fertility decline. Bagavos and Tragaki [16]; Westoff, Bietsch and Koffman [10] as well as Shakya and Gubhaju [17] also observed that increasing women’s educational attainment is a key factor contributing to sustained fertility decline. However, this finding partly disagrees with Cai [18] who in a study conducted in China found that improvement in education had no effect on fertility change. This may partly be due to socioeconomic context differences between Uganda and China as well as the different policies that exist in the two countries. In China, the strong government intervention in birth control policy could have suppressed variation in fertility. This is not the case for Uganda since the number of children is largely a personal choice and this can partly explain why education is such a significant contributor to changes in Uganda’s fertility. Our findings also point to the need for mechanisms to increase the age at which women marry. The results are in line with other studies that contended that an increase in age at first marriage reduces fertility [5, 19, 20]. This finding also concurs with Beatty [1] who contended that fertility transition is not likely to begin in a country where age at first marriage for women is still low.

An increase in the proportion of women that were exposed to family planning messages between 2006 and 2011 was found to have contributed to the variation in fertility during the period. The finding suggests that if the population experienced increased exposure to family planning messages, a fertility transition can be facilitated. Exposure to family planning messages may lead to changes in attitudes towards large families and use of contraceptive methods which in turn lead to adoption of small family norms such as contraceptive use. Appropriate mass media campaigns on family planning should target high fertility areas rural areas. The importance of exposure to mass media has been reported to be a determinant of the number of children desired and increased use of modern contraceptives [8, 10, 21]. Although contraceptive use among women in Uganda was still low, our findings indicated that contraceptive use contributed significantly to the change in number of children ever born. This finding points to the need for continued and increased government and international support for quality family planning if sustainable fertility reduction is to be achieved. This finding confirms what numerous studies have asserted about contraceptive use significantly driving fertility transition [9, 12, 17, 19, 20, 23]. However one study conducted in Uganda reported that modern contraceptive use did not influence the country’s fertility rates [5]. This may be because the study by Kabagenyi et al. looked at contraceptive use for one survey period but did not cover the time variations in the effects of contraceptive use.

The contribution of family size preference to the observed change in fertility can be linked to the reduction in the proportion of women desiring large family size (at least five children). Family size preferences affect people’s fertility behaviors and especially decisions on whether to use or not to use contraceptives. There is need to continue reaching the population especially in rural areas with information about the benefits of smaller families. The importance of shift in desired family size in fertility decline was confirmed in various studies [8, 10, 12, 22, 23]. In fact an earlier study asserted that fertility desires and not contraceptive access matter in fertility change [19].

This study also found that increase in the proportion of women who delayed their sexual intercourse to at least 20 years influenced the observed variation in children ever born. Delayed sexual intercourse implies delayed exposure to pregnancy and childbearing. Government and other stakeholders such as parents, local leaders, and religious leaders should keep encouraging young people to delay entry into sex. Most of the sexual abstinence messages in Uganda have focused on the prevention of sexually transmitted infections and especially the Human Immune-deficiency Virus (HIV), it is thus important that such messages incorporate pregnancy and childbearing.

The increases in the proportion of women residing in urban areas significantly influenced the 2006–2011 observed change in fertility. This may be due to improved access to family planning services and information, education and existence of smaller family size norms that usually characterize urban areas. This finding resonates with other studies that have found faster change in fertility among women residing in urban areas compared to rural counterparts [5, 10, 12, 17, 20]. Relatedly, the findings have indicated that if household wealth improved, fertility decline. The government of Uganda initiated the “Operation wealth creation” which if well implemented presents an opportunity for the country to achieve faster fertility decline especially in the rural areas where they are based. Dribe, Hacker and Scalone [24] support this as they contended that middle classes and the rich class experience faster fertility transition compared to the poor.

Although the time period for this analysis is very short for detailed explanation of demographic transitions which are known to take longer periods, the two survey years chosen, 2006 and 2011 represented a period in which visible change in fertility was reported. The 2006 and 2011 Uganda Demographic and Health Survey reports indicated that the fertility rate in Uganda reduced from 6.7 children per woman in 2006 to 6.2 children per woman in 2011 [15]. Earlier surveys had indicated that fertility had persisted just over 6.7 children per woman. The study intended to identify the factors that contributed to the observed change in fertility between 2006 and 2011.

In our analysis, we only included women who had ever had sex. Most studies on fertility focus on married and ever married women or all women of reproductive age. By focusing on ever married and married women, such analyses exclude non-marital and premarital fertility which are seemingly increasing in recent times. The current study focused on women who had ever had sex so that only women exposed to the risk of pregnancy and childbirth are included in the analysis. However, this inclusion criteria may represent a limitation of the study as there may have been under reporting or even refusal to report on sexual activities especially among adolescents who may fear to disclose freely disclose their sexual histories. In most cultures, unmarried young people are expected to abstain from sexual intercourse and thus such young people who are not married may decline to disclose their sexual activity status.

There is a possibility that some women who were and interviewed in the 2006 survey were again interviewed in 2011. Even if some women were interviewed both in 2006 and 2011, there could have been changes in characteristics as well as reproductive behavior. For instance, a woman who was interviewed as a non-user of family planning in 2006 could have been interviewed as a “user” in 2011. Relatedly, a woman who was interviewed aged 20 years and who had not yet given birth in 2006 was aged 25 years in 2011 and could have even given birth.

There may be rural and urban disparities in the importance of the factors explored by this study. We propose that future studies explore the determinants of change in the rural and urban areas separately in order to understand the factors influencing fertility change in the two areas.

The strength of this manuscript is that the analysis is based on survey data which is nationally representative. The analysis technique used facilitates the portioning of change in an outcome over time into components attributable to changing socioeconomic and demographic characteristics of women and changing reproductive behaviors.

## Conclusion

The decomposition technique quantified the contribution of changing characteristics and changing reproductive behavior of the women to the observed change in fertility pattern among women in 2006 and 2011. The key contributors to the change in fertility were; changes in age at first marriage, age of women, education level attained, ideal number of children, exposure to family planning messages, age at sexual debut, place of residence, wealth index and contraceptive use.

As Uganda continues to focus on harnessing its demographic dividend resulting from changes in the age structure of the population emanating from rapid fertility decline, it is important that the government continues its support for investments in education and wealth creation programs. The findings point to the need for government and its partners to increase the number of family planning service points and intensified outreaches focusing on fertility control. Increasing support for family planning activities and especially efforts to ensure increased availability and accessibility of quality family planning methods and the intensification of mass media campaign efforts to provide messages on the benefits of family planning and fertility limitation will not only contribute to utilization of contraceptives but would also lead to changes in attitudes towards large families.

## Abbreviations

ANOVA: analysis of variance
CDC: Centers for Disease Control and Prevention
CEB: Children ever born
DHS: Demographic and Health Survey
IRR: Incident Rate Ratio
MCEB: Mean Children Ever Born
TFR: Total Fertility Rates
UBOS: Uganda Bureau of Statistics
UDHS: Uganda Demographic and Health Survey
